# Reduction of Haematite Using Hydrogen Thermal Plasma

**DOI:** 10.3390/ma12101608

**Published:** 2019-05-16

**Authors:** Masab Naseri Seftejani, Johannes Schenk, Michael Andreas Zarl

**Affiliations:** 1 Department of Metallurgy, Montanuniversitaet Leoben, 8700 Leoben, Austria; Johannes.Schenk@unileoben.ac.at (J.S.); Michael-Andreas.Zarl@unileoben.ac.at (M.A.Z.); 2 K1-MET GmbH, Stahlstraße 14, A-4020 Linz, Austria

**Keywords:** hydrogen plasma, smelting reduction, iron oxide, plasma arc, degree of hydrogen utilization, degree of reduction, haematite, basicity

## Abstract

The development of hydrogen plasma smelting reduction as a CO_2_ emission-free steel-making process is a promising approach. This study presents a concept of the reduction of haematite using hydrogen thermal plasma. A laboratory scale and pilot scale hydrogen plasma smelting reduction (HPSR) process are introduced. To assess the reduction behaviour of haematite, a series of experiments have been conducted and the main parameters of the reduction behaviour, namely the degree of hydrogen utilization, degree of reduction and the reduction rate are discussed. The thermodynamic aspect of the hematite reduction is considered, and the pertinent calculations were carried out using FactSage^TM^ 7.2. The degree of hydrogen utilization and the degree of reduction were calculated using the off-gas chemical composition. The contribution of carbon, introduced from the graphite electrode, ignition pin and steel crucible, to the reduction reactions was studied. The degree of reduction of haematite, regarding H_2_O, CO and CO_2_ as the gaseous reduction products, was determined. It is shown that the degree of hydrogen utilization and the reduction rate were high at the beginning of the experiments, then decreased during the reduction process owing to the diminishing of iron oxide. Conducting experiments with the high basicity of slag B_2_ = 2 led to a decrease of the phosphorus concentration in the produced iron.

## 1. Introduction

The main reason for the development of the HPSR process is to minimize CO_2_ emissions in the iron- and steel-making processes. The iron and steel industry is responsible for approximately 5% of anthropogenic CO_2_ emissions and approximately 27% of CO_2_ emissions from the manufacturing sector in the world [1]. The intensity of CO_2_ emissions in the steel industry depends on the production route and varies between 1080 and 2150 t CO_2_/t crude steel [2].

Around 70% of total steel is produced by the blast furnace (BF)-basic oxygen furnace (BOF) integrated route. The electric arc furnace (EAF) route, for which scrap and direct reduced iron (DRI) or hot metal are used as input materials, accounts for the production of 30% of global steel [3]. Around 0.3% of crude steel production is produced by the smelting reduction processes such as COREX. The DRI processes are categorized in two groups based on the use of reducing agent, natural gas-based direct reduction (DR) processes like MIDREX, HYL and Finmet, and coal-based DR processes like FASTMET and Comet [4]. Natural gas-based DR processes with the integration of EAF are the least CO_2_ emitting processes among conventional iron and steel making routes.

To produce one ton of liquid steel by the integrated BF-BOF route, 2120 kg and by the paired HYL 3-EAF 1125 kg of CO_2_ is emitted [5]. For the recycling of 100% scrap by EAF, 466 kg CO_2_/t liquid steel is produced [6]. However, considerable efforts have been taken to determine opportunities for the reduction of CO_2_-emissions in the iron and steel industry [7,8]. Potential sources of carbon for the generation of CO_2_ in EAF are graphite electrode, scraps and charged material. Graphite electrode consumption in EAF is in a range of one and 10 kg/t of liquid steel, which depends on the quality of electrode and the operation conditions. The graphite electrode consumption in Germany in 2010 was approximately 1.1 kg/t of liquid steel [9].

A detailed description of the thermodynamic concepts of iron oxides reduction using hydrogen thermal plasma is given in the other work [10] by some of the present authors. In HPSR, the first step of the reduction reaction of haematite by hydrogen in the plasma state is given by:

(1) $${\mathrm{Fe}}_{2}O_{3}+2\mathrm{Hydrogen}\;\mathrm{plasma}\;\left(2H,\;2H^{+},{\;H}_{2}^{+},\;2/3H_{3}^{+}{\;\mathrm{or}\;H}_{2}^{*}\right)↔\mathrm{FeO}+2H_{2}O\;\left(g\right)$$

The second step is the reduction reaction of FeO to produce metallic iron

(2) $$\mathrm{FeO}+\mathrm{Hydrogen}\;\mathrm{plasma}\;\left(2H,\;2H^{+},{\;H}_{2}^{+},\;2/3H_{3}^{+}{\;\mathrm{or}\;H}_{2}^{*}\right)↔\mathrm{Fe}+H_{2}O\;\left(g\right)$$

The Ellingham diagram for metal-oxide and H_2_O–H_2_, H_2_O–H and H_2_O–H^+^ lines over temperature indicate that the H_2_O–H^+^ line lies below the other lines; consequently, hydrogen in the plasma state can reduce all metal oxides [11,12,13].

HPSR has been introduced as a CO_2_-free iron- and steel-making route at Montanuniversitaet Leoben. Several researchers [14,15,16,17,18,19,20,21] at the Chair of Ferrous Metallurgy have studied the reduction behaviour of different iron oxides using hydrogen, carbon, carbon monoxide, and natural gas in the plasma state.

Weigel et al. [22,23] carried out some experiments to reduce iron ores using an argon-hydrogen plasma in a DC-plasma smelting furnace. They used a thoriated tungsten electrode with a water-cooled plasma torch to conduct the experiments which caused the removal of the contribution of carbon to the reduction reactions. Because to scale up the process with a graphite electrode is more feasible than that to scale up with a water-cooled plasma torch, in the present study, a hollow graphite electrode was used. For their study, 680 g of a high-quality Samarco iron ore with a total Fe of 66.8% was used. The flow rates of the argon and hydrogen were nine and 10 L/min, respectively. They reported that the degree of hydrogen utilization ($\eta_{H_{2}}$) was between 43% and 50% with a total degree of reduction ($\eta_{tot}$) of approximately 75% in 35 min. $\eta_{H_{2}}$ was in the range of thermodynamic equilibrium, which means that there was not any restriction of the reaction rate. However, Kamiya et al. [24] presented a mechanism with seven steps for the reduction of iron oxide using hydrogen plasma. They showed that the limiting steps of the reduction control the reduction rate. 

The previous work of the authors of the present paper [25] covers the kinetics of the reduction of iron oxide using hydrogen. They reported that the reduction rate of iron oxide using hydrogen in the plasma state was greater than that of the molecular state. To compare the kinetics behaviour of hydrogen with solid carbon and CO, Nagasaka et al. [26] prepared a plot from the results of pertinent studies. In terms of the iron oxide reduction using solid carbon, the results of researchers [27,28,29,30] and using CO, the studies by researchers [29,31,32] have been assessed. As a result, the reduction rate of liquid wüstite using solid carbon depends on the temperature. At temperatures above the FeO melting point, the reduction rate significantly increased. The results of the reduction rates with CO reported by different researchers were in good agreement and they showed that the reduction rate of wüstite in the liquid state is one order of magnitude greater than that of the solid state. At the present work, not only solid carbon from the graphite electrode introduced to liquid iron oxide but also CO, formed during operation, contributed to the reduction reactions of iron oxides.

Hiebler et al. [12] introduced the concept of an HPSR industrial plant with a capacity of 1.2 Mt liquid steel per year. They compared the costs of liquid steel production using an HPSR industrial scale with a best conventional cost-optimized integrated route of steel making with a capacity of 3.2 Mt liquid steel per year. They showed that the production cost of liquid steel using HPSR is approximately 20% cheaper than that of other conventional iron and steel making processes.

More than 60% of the world’s direct reduced iron (DRI) is produced by the MIDREX process. The operation conditions are different from plant to plan which caused different productivities. The degree of hydrogen utilization in a MIDREX plant depends on process characteristics such as the inlet gas flow rate, temperature of gas, gas composition, reactor size, properties and feeding rate of lumo ore or pellets. To assess the MIDREX process and compare it with the HPSR process, the operation data of the Gilmore plant as an example was evaluated [33]. Table 1 shows the inlet gas and the off-gas composition of Gilmore plant.

The flow rate of inlet gas was 53,863 Nm^3^/h at a temperature of 930 °C and at 1.4 barg pressure to produce 26.4 t/h with the degree of metallization 92.3%. Regarding the hydrogen concentration in inlet and outlet gas composition, the degree of hydrogen utilization is approximately 29.6%.

## 2. Facilities

**Figure 1:**
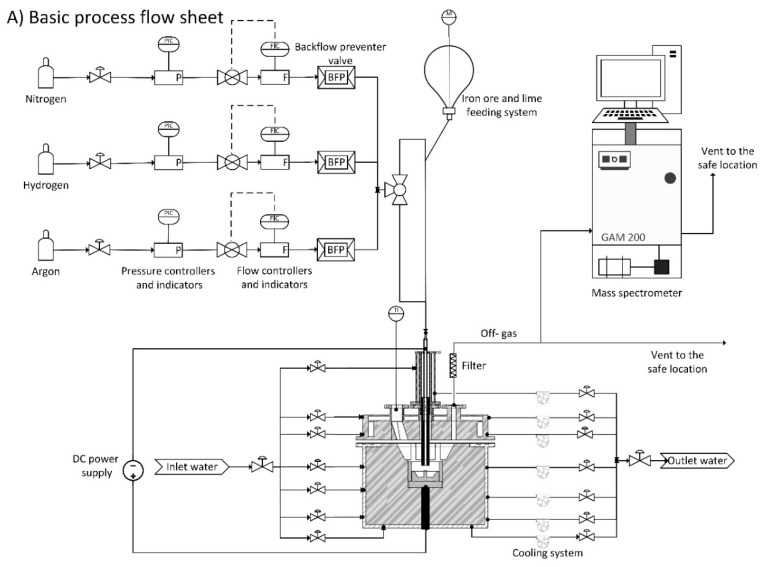

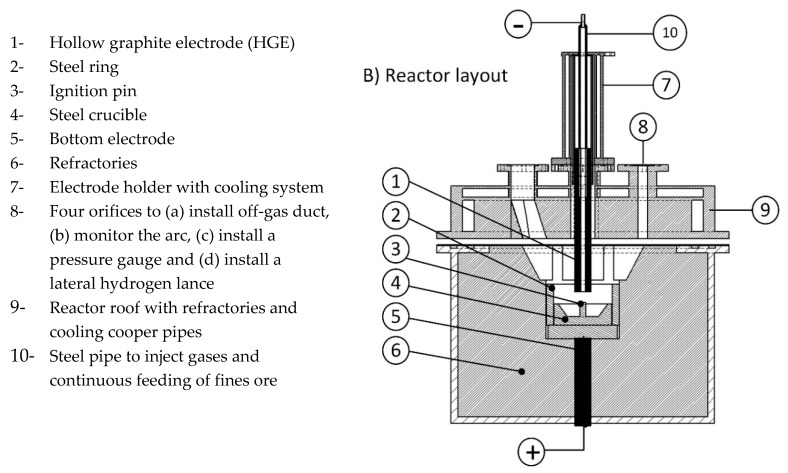
Schematic of laboratory-scale plasma facility at Montanuniversitaet Leoben: (**A**) process flow diagram and (**B**) reactor layout [10].

A laboratory-scale hydrogen plasma facility has been installed at the laboratory of the Chair of Ferrous Metallurgy in Montanuniversitaet Leoben as an early-stage means to assess the characteristics of the new technology. In order to obtain highly reliable experimental results and move closer to the actual industrial scale, the plasma experimental facility has recently been redesigned, and new instruments and equipment have been installed. Figure 1 shows the schematic of the renewed plasma experimental equipment.

**Figure 2:**
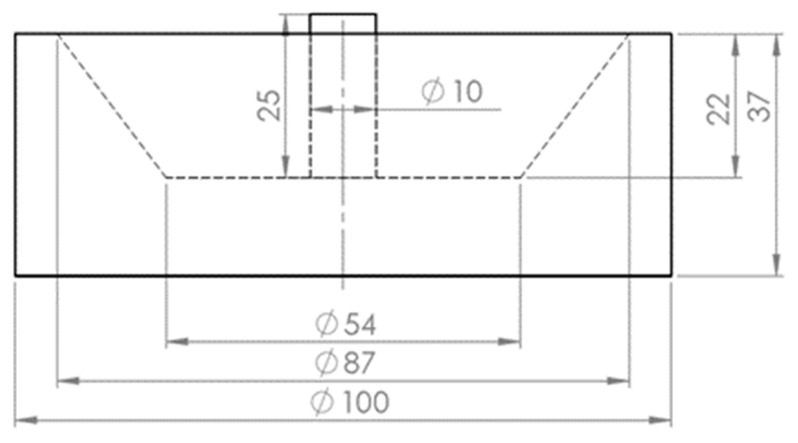
Drawing of the steel crucible, dimensions are in mm.

**Figure 3:**
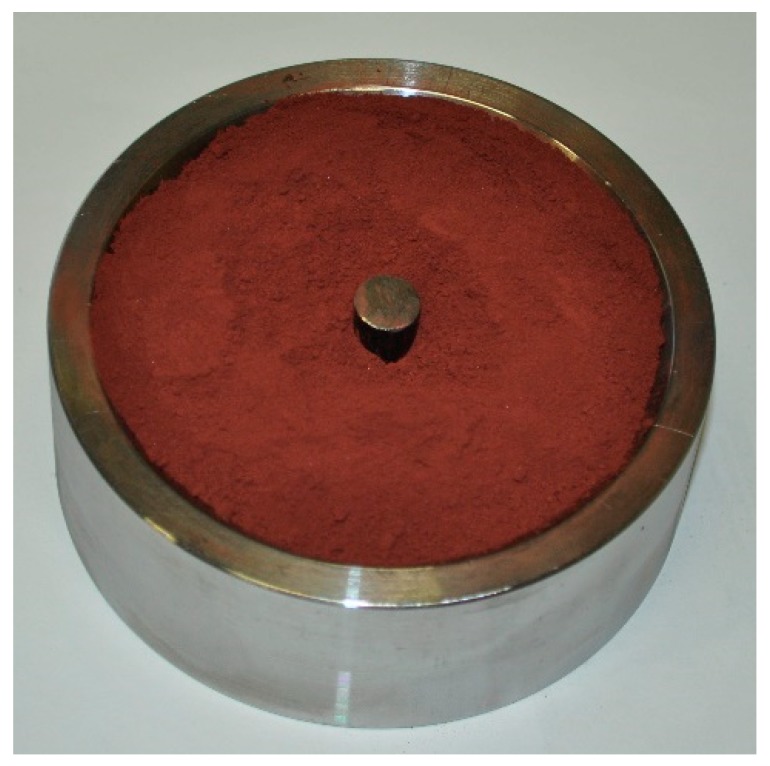
Steel crucible with iron ore.

In this process, the plasma arc was generated between the tip of the hollow graphite electrode (HGE) as the cathode, and the ignition pin, located in the steel crucible, as the anode. The outer and inner diameter of the electrode were 28 and 8 mm, respectively. The mode of the arc attachment was DC transfer. The power supply provided a maximum electric power of 8 kW, with a voltage between 20 and 100 V and a maximum current of 150 A. However, a power of approximately 6 kW with a current of 100 A and voltage 60 V was used for the normal operation, considering an arc length of 35 mm. Therefore, the arc volts/length for the normal operation was 1.7 V/mm. A steel crucible of maximum 200 g capacity and with the weight of 1606 g was used which was electrically connected to the bottom electrode. To ignite the arc, an ignition pin with a diameter of 10 mm was welded on the middle of the crucible to start arcing. Figure 2 shows the drawing of the steel crucible and Figure 3 is a photo of the steel crucible filled with the powdered iron ore.

Argon or nitrogen can be used as a plasma gas and hydrogen as a reducing agent. However, in this study, only argon has been used to be mixed with hydrogen. A mixture of gas containing argon and hydrogen was injected into the plasma arc zone through the HGE. Considering the high temperature of the plasma arc, this design provided the best conditions for atomization and ionization of the hydrogen particles. There were four different orifices on the roof of the vessel. One orifice was employed to install an optical spectrometer to monitor the light from the arc. The second orifice was used for the off-gas analysis. The third orifice was used to install a manometer to measure the pressure, and through the fourth one a lance could be applied for the injection of lateral hydrogen. The plasma vessel and the electrode holder were equipped with a water-cooling system to avoid heat penetration from the heating sections. A steel grid, glass wool, water bottle and molecular sieve were used in the off-gas cleaning system to collect the dust and remove the water vapor. A mass spectrometer, GAM 200 produced by InProcess Instruments Gesellschaft für Prozessanalytik mbH, was used to analyse the chemical composition of the off-gas.

The plasma laboratory was also equipped with a continuous feeder to feed the mixture of iron ore and additives to study the reduction behaviour of the iron ore. However, in the present work, it was not used.

To obtain enough knowledge and recommend parameters to design an HPSR industrial plant, a pre-commercial plant should be employed. Trials with a pilot plant generate not only more detailed mass and energy balance data, but also a better estimation of the degree of hydrogen utilization and the reduction rate of iron oxides. Therefore, an HPSR pilot plant is under construction in voestalpine Stahl Donawitz GmbH.

The use of a steel crucible to conduct the experiments resulted in the freezing of parts of the liquid oxides, which caused a decrease in the reduction rates and the degree of reductions. Therefore, refractory material was used for the pilot plant.

## 3. Experimental Procedure

### 3.1. Materials and Sample Preparation

In this study, the reduction behaviour of haematite using a pre-mixture of H_2_–Ar plasma arc was assessed. The experiments were carried out with three different weights of iron ore powder in steel crucibles. The powders were melted and reduced by hydrogen to study the parameters that influence the iron ore reduction behaviour. Table 2 shows the experimental program with the definition of the experiment parameters.

For the reduction of iron ore, a premixed 50% H_2_–50% Ar gas with a total flowrate of 5 L/min for all runs was used. The flow controllers could supply a mixture with different hydrogen-to-argon ratios and different flow rates.

Carajas iron ore as a high-quality raw material was selected to run the experiments. The chemical composition of Carajas iron ore is shown in Table 3.

However, there was a possibility to feed the iron ore continuously with a continuous feeding system, and in this study the total amount of iron ore was charged into the crucible before the test run. The steel crucible was located on a steel disc connected to the 4-pin electrodes. One steel ring with a layer of MgO refractory was located on the outer diameter of the crucible to protect the reactor side-wall from the plasma arc radiation. Then, the roof was assembled and the whole system was completely sealed. To adjust the slag basicity, lime with the same grain size distribution was used. Grain size distribution of lime and iron ore were separately classified, and then mixed. Table 4 shows the grain size distribution of the Carajas iron ore and lime.

To remove the gases produced due to the lime calcination in the plasma reactor, it was calcined at the temperature of 1100 °C before mixing with iron ore powder. To prepare the sample powder, 3.4 g of calcined lime with 97% of CaO and 2.2% of MgO content was mixed with 100 g of iron ore to reach the basicity of two.

Not only the ignition pin but also the steel crucible were melted during pre-melting and were mixed with the iron oxide liquid. Therefore, the carbon content of those steel parts contributed to the reduction reactions. The crucible was partially melted, so it was essential to assess the melting of the crucible after each test run to determine the amount of carbon contributed to the reduction reactions from the steel crucible. The crucible was cut from the middle and analysed using the spectrometer, and the micro- and macrostructure were evaluated using an optical microscope. The partially melted crucible from each test run was observed and the weight was approximately calculated. The other source of carbon introduced to the melt was from the ignition pin. The weight of the ignition pin was 15 g. The chemical composition of the ignition pin and the steel crucible is shown in Table 5.

One HGE as a cathode with an outer diameter of 26 mm and inner diameter of 8 mm was used to inject the premixed gas through it. By means of a glass window installed on one of the orifices on the reactor roof, the plasma arc was monitored.

The off-gas contained dust, mainly carbon from graphite electrode. Dust creates problems for the mass spectrometer (MS), so it should be appropriately cleaned before entering the MS. Therefore, a steel grid, glass wool, coolant/water trap, molecular sieve 3A° type 562 C and silica gel were used for the off-gas cleaning. This off-gas cleaning system setup can not only capture the accompanying dust but also remove water vapour from the off-gas. The off-gas was analysed using a mass spectrometer GAM 200 during the operations. The MS was calibrated by a calibration gas which the chemical composition is shown in Table 6. The calibration gas was produced to meet the requirements of Grade 1. Therefore, the relative uncertainty for the components with a content between 0.1% and 4.9% is ±2% and for those more than 4.9% is ±1%.

### 3.2. Description of the Operation

Prior to the ignition of the plasma arc, the whole system was purged by argon with a flow rate of 5 L/min for 10 min to withdraw oxygen from the system. While purging, the chemical composition of the outlet gas was monitored in the MS to be certain of decreasing the oxygen percentage to less than 1%. Then, the arc was ignited and pure argon with a flow rate of 5 L/min was used for pre-melting to make a liquid pool of iron ore inside the steel crucible. This step of the operation was run for 3 min, and then the injection gas switched from pure argon to premixed hydrogen/argon with a flow rate of 2.5/2.5 L/min to begin the reduction process.

During the reduction step, the gas flowrate and the composition were kept constant. The voltage was not constant during the reduction step due to the changes of hydrogen concentration. At the beginning of the reduction process, the degree of hydrogen utilization was high, which caused a decrease in the hydrogen concentration. Then the degree of hydrogen utilization decreased continuously during the reduction process, and it led to a higher energy consumption and an increase of voltage. Because hydrogen is a diatomic molecule and needs more energy than argon for the ionization [34,35,36], when the voltage exceeded 100 V, it caused a cut off of the plasma arc. To prevent the operation shutting down, the arc length, the distance from the tip of the electrode and the surface of the molten metal were all readjusted. Hence, corresponding to the voltage, the HGE was manually driven downwards to decrease the arc length. 

The reduction step lasted until $\eta_{H_{2}}$ reached less than 15% which is a low degree of hydrogen utilization. At the lower degrees, the arc mainly runs between the graphite electrode and the edge of the steel crucible or on the already reduced sections, therefore, the operation was stopped. After completing the reduction process, hydrogen injection was stopped, and the reactor was purged by nitrogen with a flow rate of 5 L/min to remove hydrogen from the system. To conduct the mass balance, the crucible, refractory ring, electrode and filters were weighed before and after each experiment. To assess the reduction process and the chemical composition of the steel products, slag was separated from the crucible, and then the crucible was cut from the middle and analysed using a spectrometer.

### 3.3. Method of Calculation of Hydrogen Utilization Degree and Degree of Reduction

To assess the data and to obtain results with the minimum amount of deviation, the raw chemical composition shown by MS should be corrected in two steps. The first step is to remove the unwanted elements from the chemical composition, namely nitrogen, oxygen and water. After removing water from the off-gas, less than 0.2% water is still shown by MS as remaining in the off-gas. Small amounts of nitrogen (in a range between 0.2% and 0.5%) and oxygen (less than 0.06%) were also shown in MS. The reason was the sucking of air by the MS from the outlet pipe because the off-gas flow rate and pressure were too low. In order to reduce the errors, the off-gas composition was corrected by removing H_2_O, N_2_ and O_2_. The second step is to remove the deviation of the MS. For this reason, after calibration of the MS, the calibration gas was again analysed by MS. Even after this calibration, the composition of the off-gas shown by MS was not exactly the same as its actual composition. This means that there was a deviation in the chemical composition shown by MS. Therefore, to eliminate the MS deviation, the results of the MS were corrected by the deviation factors of each element, which were obtained from the difference between the real calibration gas composition and the measured values. Table 7 shows the deviation of each element.

The deviation of MS by this method was removed from the results. Therefore, only the relative uncertainty of each component should be considered.

The measurement cycle by MS was set to 8.4 s, which was enough time to deliver reliable results for the gas composition changes during operations.

Regarding the premixed argon/hydrogen inlet gas and the reduction reactions, the off-gas released from the reactor comprises:

1. Ar: argon was the unreacted gas and can therefore leave the reactor without any reaction.
2. H_2_: a significant amount of hydrogen left the reactor without any reaction due to the thermodynamic equilibrium and kinetics limitations.
3. H_2_O: water vapor was the product of the reduction reactions of metal oxides with hydrogen.
4. CO and CO_2_: these gases were the products of the reduction of metal oxides by carbon. The erosion of the graphite electrode and carbon from melting of the ignition pin and steel crucible caused carbon to enter the iron oxide melting pool. The carbon then reduced the liquid iron oxide and CO and CO_2_ were released.

**Figure 4:**
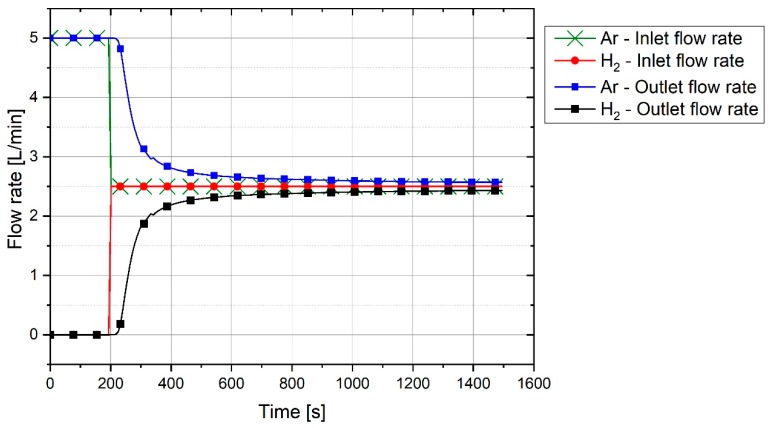
Reference flow rate of a premixed 50% Ar/50% H_2_ gas with a total flow rate of 5 L/min.

The off-gas flow rate was not equal to the inlet gas flow rate due to the formation of CO and CO_2_ in the reactor and the condensation of H_2_O in the off-gas cleaning system. To define the total flow rate and accordingly the flow rate of each gas, the flow rate of argon was used as a reference. Argon was an unreacted element and left the plasma reactor without any reaction. However, the outlet flowrate of argon was not the same as the inlet flowrate. To obtain the reference flow rate, argon and hydrogen were injected into the reactor without arcing to simulate the flow of off-gas. Figure 4 shows the graph of outlet gas composition after switching the gas from pure argon to a mixture of 50% argon and 50% hydrogen with a total flow rate of 5 L/min. This gas composition and the flowrate were used for all three experiments.

The plot shows that it takes 100 s for hydrogen to reach the MS. With the passage of time, argon was replaced by hydrogen. It is seen that, even after 1200 s, the composition of the outlet gas was not the same as that of the inlet gas. Therefore, it took time for the chemical composition of the inlet gas and the outlet gas to become closer. Hence, this graph was used as a reference to compare the results of the experiments and to find the real amount of hydrogen and water vapor produced.

For the experiments, before the start of arcing, the plasma reactor was purged with argon so that only argon was inside the reactor. Pre-melting was done by flowing pure argon, and for the reduction process, the gas was switched to a hydrogen/argon mixture. To calculate the degree of hydrogen utilization, the difference of hydrogen concentration between the reference flow rate and the MS result was considered.

Corresponding to the amount of argon from the reference, the total flow rate was defined by:

(3) Total flow rate [L/min]=Ar outlet flow rate [L/min]/(Ar in the off gas [%])×100

From the total flow rate, the flow rate of each gas was calculated. Water vapor was condensed in both the off-gas duct and the cleaning system. Hence, it was not possible to calculate the flow rate of the water vapor directly from the off-gas composition. Therefore, from the difference between the hydrogen flow rate in the off-gas and in the reference flow rate at the same time, the water vapor was calculated.

(4) $$\begin{array}{cc} H_{2}O\;\mathrm{flow}\;\mathrm{rate}\;\left[L/\min\right] & =\left(H_{2}\;\mathrm{flow}\;\mathrm{rate}\;\mathrm{in}\;\mathrm{the}\;\mathrm{off}-\mathrm{gas}\;\left[L/\min\right]\right)\\ & -(H_{2}\;\mathrm{flow}\;\mathrm{rate}\;\mathrm{from}\;\mathrm{reference}\;\mathrm{flow}\;\mathrm{rate}\;\left[L/\min\right]) \end{array}$$

Finally, the total flow rate regarding water formation was calculated and the chemical composition of the off-gas was defined. $\eta_{H_{2}}$ is the hydrogen utilization and was accrued from the H_2_ and H_2_O amount by

(5) $$\eta_{H_{2}}\left[\%\right]=\frac{{\%H}_{2}O}{\left({\%H}_{2}+{\%H}_{2}O\right)}\times 100$$

The reduced amount of oxygen was calculated by summing up the amount of oxygen in H_2_O, CO and CO_2_ by

(6) $$m_{O,H_{2}O}=\overset{n}{\underset{C=1}{\sum }}\left[\frac{H_{2}O\;\mathrm{flow}\;\mathrm{rate}\;[L/\min]}{22.4}\times 16\times \left(C_{n}-C_{n-1}\right)\left(s\right)/60\right]\left[g\right]$$

(7) $$m_{O,\mathrm{CO}}=\overset{n}{\underset{C=1}{\sum }}\left[\frac{\mathrm{CO}\;\mathrm{flow}\;\mathrm{rate}\;[L/\min]}{22.4}\times 16\times \left(C_{n}-C_{n-1}\right)\left(s\right)/60\right]\left[g\right]$$

(8) $$m_{O,{\mathrm{CO}}_{2}}=\overset{n}{\underset{C=1}{\sum }}\left[\frac{{\mathrm{CO}}_{2}\;\mathrm{flow}\;\mathrm{rate}\;[L/\min]}{22.4}\times 32\times \left(C_{n}-C_{n-1}\right)\left(s\right)/60\right]\left[g\right]$$

(9) $$m_{O,\mathrm{tot}}=\left(m_{O,H_{2}O}+m_{O,\mathrm{CO}}+m_{O,{\mathrm{CO}}_{2}}\right)\left[g\right]$$

where m_O,tot_ is the total mass of oxygen, $m_{O,H_{2}O}$, $m_{O,\mathrm{CO}}$ and $m_{O,{\mathrm{CO}}_{2}}$ are the mass of oxygen in H_2_O, CO and CO_2_ espectively, and C_n_, C_n−1_ (S) are cycles n and n − 1 from MS, respectively. The degree of reduction (R_Degree_), which is the oxygen reduced by carbon and hydrogen during the experiment, was calculated by

(10) $$R_{\mathrm{Degree},{\mathrm{by}H}_{2}}=m_{O,H_{2}O}/m_{O,\mathrm{in}\mathrm{iron}\mathrm{ore}}\times 100$$

(11) $$R_{\mathrm{Degree},\mathrm{by}C}=(m_{O,\mathrm{CO}}+m_{O,{\mathrm{CO}}_{2}})/m_{O,\mathrm{in}\mathrm{iron}\mathrm{ore}}\times 100$$

Therefore, the total degree of reduction was

(12) $$R_{\mathrm{Degree},\mathrm{total}}=R_{\mathrm{Degree},{\mathrm{by}H}_{2}}+R_{\mathrm{Degree},\mathrm{by}C}$$

**Figure 5:**
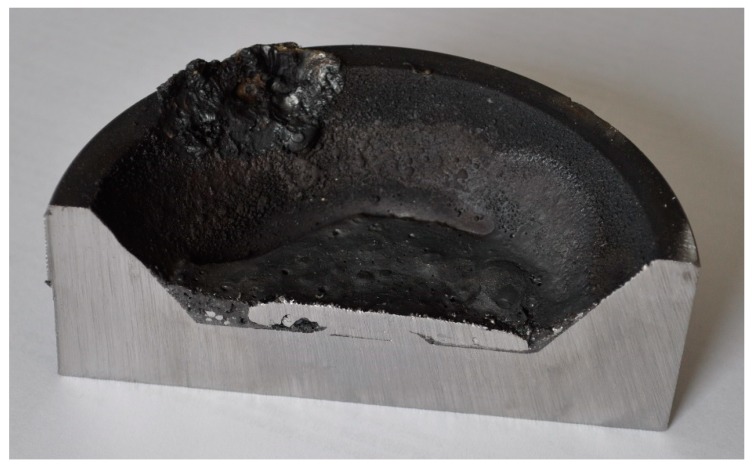
Cross-section of the crucible for Experiment 1.

Carbon was introduced into the melt from three different sources, which were the graphite electrode, ignition pin and steel crucible. Therefore, the contribution of the carbon from those sources to the reduction reactions should be taken into account. The ignition pin was completely melted and mixed with the melt. However, the steel crucible was not completely melted. The steel crucible of each experiment was cut in half and different points were analysed by a spectrometer, while the micro- and macrostructure were assessed using optical microscopy to estimate the amount of melted section. No sign of melting was observed in the crucible of Experiment 1. Figure 5 shows the cross-section of the crucible. However, the crucibles of Experiments 2 and 3 were partially melted. 

The total amount of carbon that contributed to the reduction of oxides was calculated in two ways. The first was to calculate from the chemical composition of the off-gas. The second was calculated by the following three steps:

1. The loss of graphite electrode weight by weighing before and after the experiment
2. Due to the complete melting of the ignition pin, the carbon from the ignition pin was the total carbon in the ignition pin minus the carbon remaining in the produced iron
3. Regarding the partial melting of the crucible, the carbon introduced from the crucible was the difference of the carbon content in the crucible and the produced iron multiplied by the estimated weight of the melted crucible.

The total mass of carbon obtained from weighing the parts before and after each experiment should have been equal to the total carbon calculated by the results of MS.

## 4. Results and Discussion of the Experiments

The influence of the sample weight on the degree of hydrogen utilization, degree of reduction, and reduction rate was assessed in these test runs. Three samples with weights of 50, 75 and 100 g were selected to be evaluated. First, the results of Experiment 1 are explained in detail with the relevant diagrams, and then, in the following, the results of the three experiments are presented and discussed in summary.

### 4.1. Chemical Composition of Off-Gas

**Figure 6:**
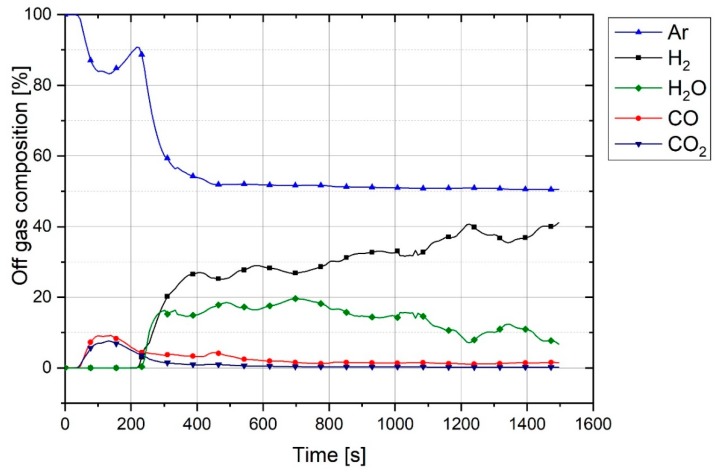
Chemical composition of off-gas from Experiment 1.

During the operation, the chemical composition of the off-gas was analysed. For instance, the off-gas composition of Experiment 1 is shown in Figure 6.

Pre-melting was done by flowing pure argon for 3 min. In this period, the off-gas comprised Ar, CO and CO_2_. CO and CO_2_ were formed due to the contribution of carbon in the reduction process. The reasons for the production of high amounts of CO and CO_2_ at the pre-melting step are the creation of thermal shock and spatters. At the beginning of the experiment, the graphite electrode was cold, and, with the generation of the arc, it was locally heated up. This phenomenon applied a thermal shock to the electrode and caused an increase of the erosion rate of the graphite electrode. The graphite-eroded particles entered the crucible and contributed to the reduction of the iron ore. The other reason is the spatter balls sticking to the electrode surface or mainly to the tip of the electrode. At the beginning of arcing, the electrode was cold, and spatters could stick easily to the graphite. With the increase of the electrode temperature, the spatter balls melted and left the electrode. The other reason is the dissociation of haematite at high temperatures, which is discussed in this chapter. Therefore, this led to the creation of a sharp peak of CO and CO_2_ at the beginning of the operation, and then the amount of CO and CO_2_ continuously decreased. However, the sticking of spatter to a cold area of the electrode caused the CO and CO_2_ lines to fluctuate.

Moreover, CO can be formed during the reduction process by the absorption of the water vapor at the graphite electrode [37]. In this case, the following reaction can occur:

(13) $$C+H_{2}O=\mathrm{CO}+H_{2}$$

**Figure 7:**
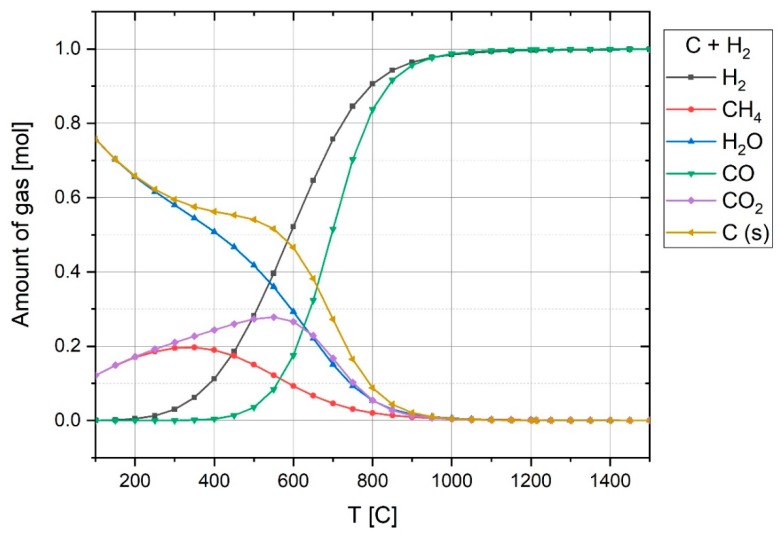
Composition of 1 mol C and 1 mol H_2_O at equilibrium at 1 atm pressure over temperature.

To assess the formation of CO and H_2_ by the above reaction, the equilibrium of 1 mol carbon and 1 mol water vapor was calculated by FactSage^TM^ 7.2 (Toronto, ON, Canada, Database: FactPS and FToxide (2018)) and the results are shown in Figure 7. In terms of the thermodynamic aspect, this process caused an increase of the H_2_ and CO in the off-gas, if the electrode temperature was high. At temperatures below 550 °C, CH_4_ and CO_2_ are the dominant products. However, the rate of CH_4_ formation at the experiment conditions was too low, so it could not be formed [38,39]. The formation of CH_4_ can be checked by MS in future works.

In Figure 6, owing to the formation of high amounts of CO and CO_2_ at the pre-melting step, the total flow rate was increased, which caused the argon concentration to decrease in the off-gas because the argon flow rate was constant. During the reduction operation, while the reduction rate and degree of hydrogen utilization decreased, the amount of hydrogen increased, and in contrast, the amount of water vapor decreased.

**Figure 8:**
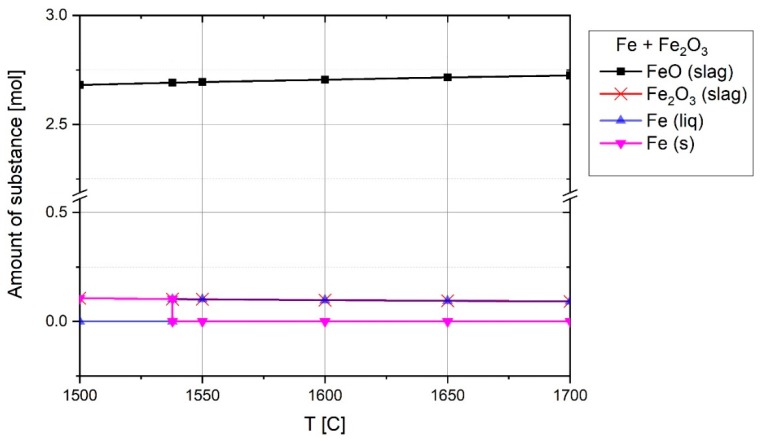
Chemical composition of 1 mol Fe and 1 mol of Fe_2_O_3_ at equilibrium at 1 atm pressure above melting temperature, FactSage^TM^ 7.2 (Database: FactPS and FToxide (2018)).

At the beginning of the experiment, the ignition pin firstly was melted due to the ignition of the arc between the ignition pin and tip of the graphite electrode. Therefore, the melted iron could react with haematite to produce wüstite. To assess the process thermodynamically, the equilibrium of iron and haematite was studied by FactSage^TM^. Figure 8 shows the chemical composition of 1 mol Fe with 1 mol Fe_2_O_3_ at equilibrium at high temperatures.

The reaction below shows the formation of FeO at 1600 °C.

(14) $${\mathrm{Fe}}_{2}O_{3}+\mathrm{Fe}=2.8\times \left(0.965\mathrm{FeO}\;\left(a=0.93\right)+0.035{\mathrm{Fe}}_{2}O_{3}\left(a=0.0015\right)\right)\left(\mathrm{Slag}\right)+0.0982\mathrm{Fe}\;\left(a=1,\;\mathrm{liq}\right)$$

**Figure 9:**
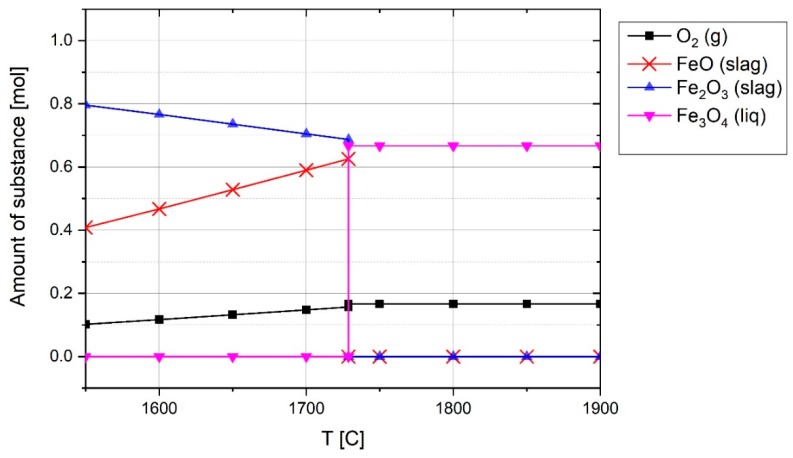
Chemical composition of 1 mol haematite over temperature at equilibrium at 1 atm pressure.

The equation at equilibrium shows that 90.18% of ${\mathrm{Fe}}_{2}O_{3}$ reacted with Fe to form FeO. Therefore, the creation of a melting pool did not cause the reduction of iron oxide to occur. However, when the arc was run between the iron ore and graphite electrode, at high temperatures, it was possible to produce magnetite. This is because, corresponding to the ${\mathrm{Fe}}_{3}O_{4}-{\mathrm{Fe}}_{2}O_{3}$ line in the Ellingham diagram and extending the line to high temperatures, the Gibbs energy will be positive [40]. Furthermore, the composition of 1 mol haematite at equilibrium at temperatures between 1500 and 2000 °C was calculated using FactSage^TM^ 7.2 (Database: FactPS and FToxide (2018)) and the results are shown in Figure 9.

Due to the high temperature of the plasma arc, haematite particles can reach this range of temperature. The reduction of haematite led to the formation of FeO and the release of oxygen. With the formation of magnetite, the FeO and Fe_2_O_3_ vanished and magnetite and oxygen were produced as the products. The pertinent reaction at the temperature of 1800 °C is written as

(15) $${\mathrm{Fe}}_{2}O_{3}=0.17O_{2}+0.67{\mathrm{Fe}}_{3}O_{4}$$

Smelting of haematite and the production of wüstite and magnetite can occur even at the reduction step because hydrogen cannot diffuse into the whole section of the melt and reduce the haematite. Therefore, if there is no hydrogen inside in a section of the melt, oxygen can be released from it. However, the released oxygen then reacts with hydrogen in the reactor volume to form water vapor, which is why an increase of oxygen was not observed during the reduction process in MS.

### 4.2. Degree of Reduction

There are two possibilities for the iron oxide reduction in the HPSR process. The first is the reduction of iron ore in liquid state. The second possibility is to reduce the powder particles of iron ore in solid state. This means that the reduction reactions can take place in the distance of the arc or melting pool. The electrical power supply was not strong enough to melt all the iron oxide particles inside the crucible or to keep the melted particles in liquid state. Therefore, the solid particles could be reduced in the presence of hydrogen. Consequently, the grain size and the size distribution of the particles were the influencing parameters on the reduction behaviour. 

**Figure 10:**
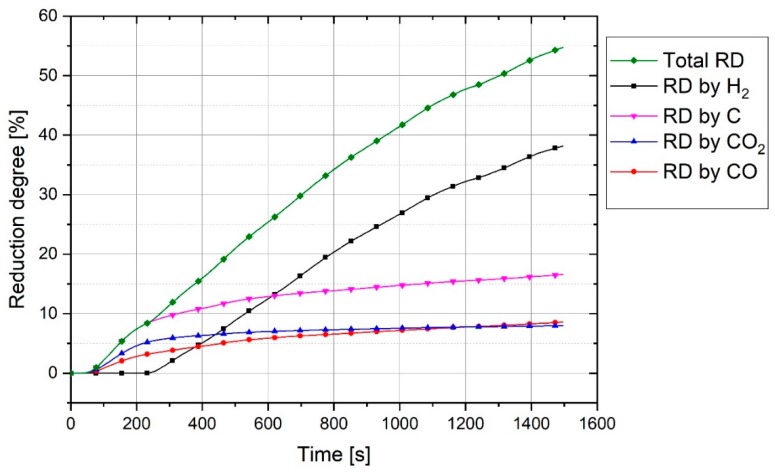
Degree of reduction by the production of CO, CO_2_ and H_2_O and total degree of reduction of Experiment 1.

The reduction behaviour was assessed by evaluating the degree of reduction, degree of hydrogen utilization and the reduction rate. Regarding the two reducing agents in the plasma reactor, hydrogen and carbon, the gas products of the reduction reductions were H_2_O, CO and CO_2_. The degree of reduction for every reductant and the total degree of reduction for Experiment 1 were calculated and the results are presented in Figure 10.

**Figure 11:**
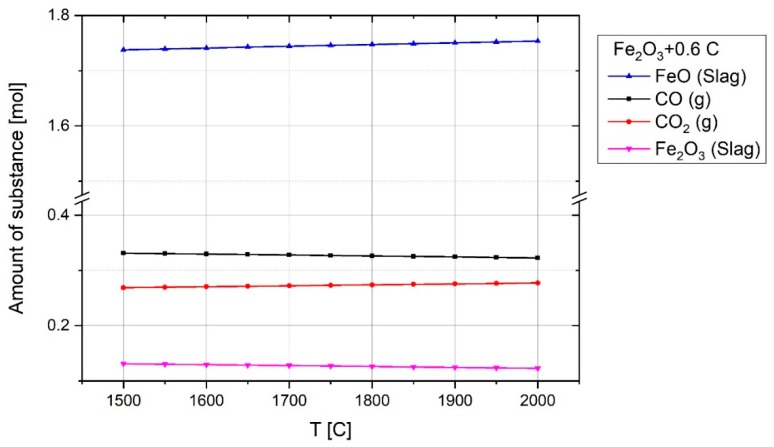
The composition of 0.6 mol of carbon and one mol of haematite at equilibrium over temperature.

The graph shows that during the pre-melting step, carbon reduced haematite to form CO and CO_2_ gases. Nevertheless, the amount of CO shown in MS was greater than CO_2_, and the oxygen removed by CO_2_ was more than that by CO. This was because every CO_2_ molecule comprises two atoms of oxygen, in contrast to one atom of oxygen in CO. For instance, at 132 s of the experiment, the amount of CO was 9.1% and CO_2_ was 7.6%, hence the ratio of CO/CO_2_ is about 1.2. To assess the results of the experiments in terms of the amounts of CO and CO_2_ in the off-gas, the stability of CO and CO_2_ was studied thermodynamically at equilibrium at temperatures between 1500 and 2000 °C using FactSage^TM^ 7.2 (Database: FactPS and FToxide (2018)). For this reason, the composition of one mol of Fe_2_O_3_ and 0.6 mol of carbon at equilibrium was calculated and the plot is shown in Figure 11.

This plot shows that at the temperature of 1600 °C, the mole fractions of CO and CO_2_ are 0.55 and 0.45, respectively. The pertinent reaction at the temperature of 1600 °C at equilibrium is given by

(16) $$0.6C+{\mathrm{Fe}}_{2}O_{3}=0.6\times \left(0.55\mathrm{CO}+0.45{\mathrm{CO}}_{2}\right)\left(\mathrm{gas}\right)+1.87\times \left(0.93\mathrm{FeO}+0.07{\mathrm{Fe}}_{2}O_{3}\right)\left(\mathrm{slag}\right)$$

Therefore, the ratio of CO/CO_2_ is about 1.2, which is in the same range shown by the MS. However, the stability of CO and CO_2_ is changed by changing the amount of carbon and haematite, and accordingly the ratio of CO/CO_2_ will be changed. For instance, the reaction between one mole of Fe_2_O_3_ and two mole of carbon at equilibrium at 1600 °C produces 0.85 mol CO and 0.15 mol CO_2_.

**Figure 12:**
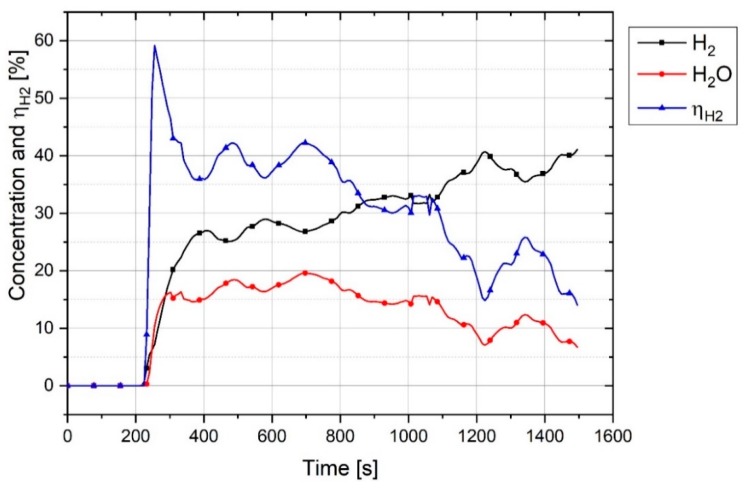
H_2_ and H_2_O concentration in off-gas and $\eta_{H_{2}}$ of the Experiment 1.

However, the erosion rate of the graphite electrode during operation depends on several parameters such as voltage, amperage and the number of spatters, the average erosion rate of the electrode under the experimental conditions was about 0.09 g/min. A large portion of the graphite electrode was consumed in the beginning of the operation. However, for a continuous process, this step cannot play an important role in terms of the CO_2_-emissions. To have a better understanding of the electrode consumption and accordingly CO_2_-emissions, the consumption of the electrode was calculated with the consideration of the average reduction rate of 0.6 g O/min and $\eta_{H_{2}}$ of 36.5%, which were average values of the reduction process (time between 300 and 800 s, Figure 12). Therefore, the electrode consumption was 31 kg/t liquid steel and amount of CO_2_-emissions was 114 kg CO_2_/t Liquid steel in the experimental process characteristics. It is expected that the electrode consumption in an industrial scale of HPSR process will be in the same range as an EAF. The only source of carbon for the direct emission of CO_2_ in an industrial scale is the consumption of graphite electrode. However, HPSR needs more energy to produce liquid iron than re-melting scraps in an EAF, which leads to increase the indirect CO_2_ emissions, which is the total CO_2_-emissions produced in previous processes such as hydrogen production.

### 4.3. Degree of Hydrogen Utilization

The degree of hydrogen utilization is the main parameter influencing the efficiency of the process which has been assessed in this study. Figure 12 illustrates the trends of the H_2_ and H_2_O concentrations during the operation and the degree of hydrogen utilization.

**Figure 13:**
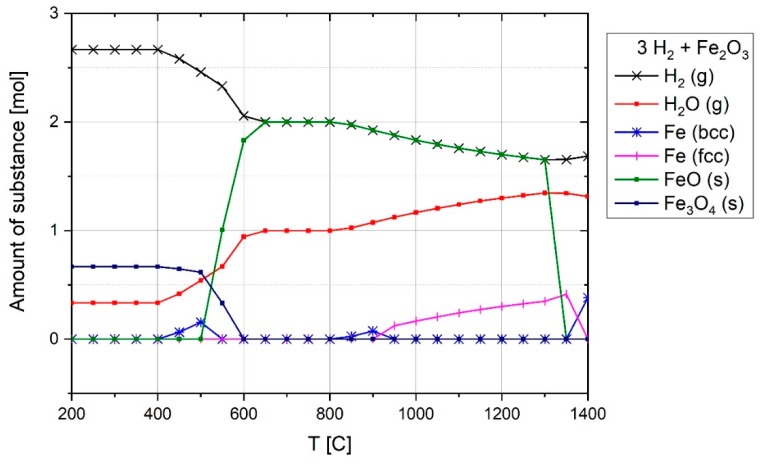
Chemical composition of 3 mol H_2_ and 1 mol Fe_2_O_3_ at equilibrium and 1 atm pressure.

$\eta_{H_{2}}$ at the start of the reduction process rose to 60%, and then fell steadily at 1450 s to less than 20%. In the beginning of the operation, the amount of hydrogen inside the reduction zone were too less, hence, $\eta_{H_{2}}$ was high. With the increase of the hydrogen inside the reactor during operation, $\eta_{H_{2}}$ decreased. The other reason for the high $\eta_{H_{2}}$ in the beginning was the reduction of trivalent iron which can be described by an overview of the operation and the melting of iron ore. The plasma reactor and the related components were cooled by the water-cooling system during operation. A maximum power of 8 kW supplied by the electric power supply was not enough to melt all the fines mixture in the crucible due to the cooling of the system by water. Therefore, the powder mixture was melted only at the arcing area and the rest of the material was in solid state. This led to applying a temperature gradient to the haematite particles inside the crucible. Haematite is capable of being reduced to wüstite even at low temperatures in the presence of hydrogen. Figure 13 shows the reduction of Fe^3+^ to form Fe^2+^ with the calculation of 3 mol H_2_ with 1 mol of Fe_2_O_3_ at equilibrium, calculated by Factsage^TM^ 7.2 (Database: FactPS and FToxide (2018)).

The thermodynamic calculation results shown in the plot illustrate that hydrogen reduces trivalent iron even at low temperatures. For instance, at 700 °C,

(17) $${\mathrm{Fe}}_{2}O_{3}+3H_{2}=3\times \left(0.67H_{2}+0.33H_{2}O\right)+2\mathrm{FeO}$$

Furthermore, to validate the results calculated by FactSage^TM^ 7.2 and shown in Figure 13, the stable phases of iron oxides in the presence of H_2_ and H_2_O at different ratios and temperatures were compared using a Baur–Glaessner diagram and were found to be in good agreement [41].

Figure 12 shows fluctuations in the degree of hydrogen utilization during operation. The main reason for the fluctuation was the position of the arc. The plasma arc could be run between the iron oxide and the tip of the graphite electrode or between the graphite electrode and the side-wall of the steel crucible. When the arc was melting the edge of the crucible, the molten bath was mainly from steel and not from iron oxide. Therefore, it caused a decrease in the degree of reduction and accordingly the degree of hydrogen utilization. Another reason for the fluctuations in the degree of hydrogen utilization was the arc created between the graphite electrode and a previously reduced section of iron oxide. This means that when one section of iron oxide in the steel crucible had already melted and reduced, then the arc was running again in this location and the reduction rate and accordingly the degree of hydrogen utilization would decrease due to the diminishing of iron oxide in the melt.

### 4.4. Influence of Sample Weight on the Reduction Behavior

**Figure 14:**
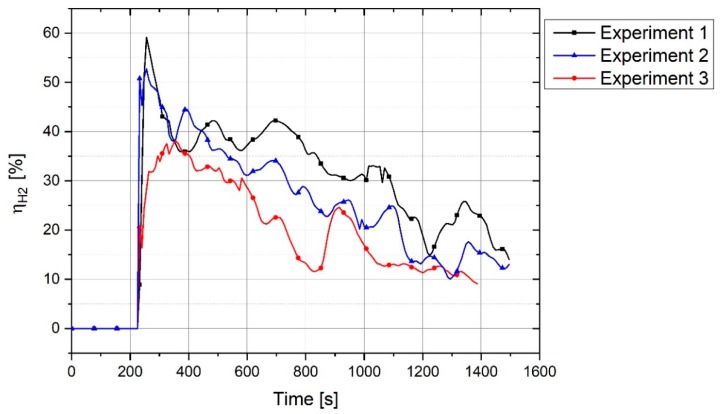
$\eta_{H_{2}}$ of Experiments 1, 2 and 3.

Figure 14 shows the degree of hydrogen utilization in Experiments 1, 2 and 3. Apart from the sample weight, all other parameters of the trials were kept constant for these three experiments. In addition, $\eta_{H_{2}}$ of Experiment 1 is higher than that of the two others due to the existence of more iron ore in the crucible. At the start of the process, $\eta_{H_{2}}$ can reach approximately by 60%, but then decreases during the reduction process. $\eta_{H_{2}}$ after 1450 s is approximately less than 20%. Experiment 3 was done with 50 g of material, which resulted in the minimum degree of $\eta_{H_{2}}$. The fluctuation of $\eta_{H_{2}}$ during Experiment 3 was greater than in the others due to the generation of the plasma arc between the electrode and steel crucible, which caused a decrease in $\eta_{H_{2}}$ and, accordingly, in the reduction rate. Because the crucible was not completely filled by iron oxide, owing to the lower resistivity of steel in contrast to iron oxide, the arc tended to run on the side of the crucible. 

**Figure 15:**
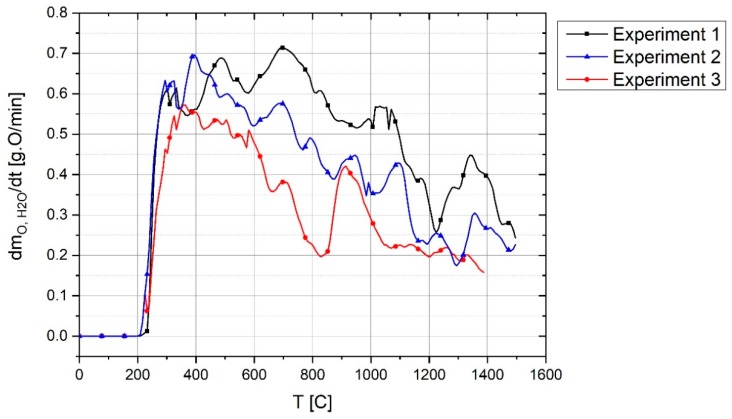
Reduction rate by hydrogen in Experiments 1, 2 and 3.

In Figure 15, the reduction rates by hydrogen in Experiments 1, 2 and 3 are compared. The changes and the reasons for them are similar to those described for the $\eta_{H_{2}}$ diagram (Figure 14). The maximum reduction rate was 0.7 g oxygen per minute and the experiments extended until reaching approximately 0.2 g O/min. Owing to the lack of iron oxide for Experiment 3, the reduction rate was lower than in the other experiments.

**Figure 16:**
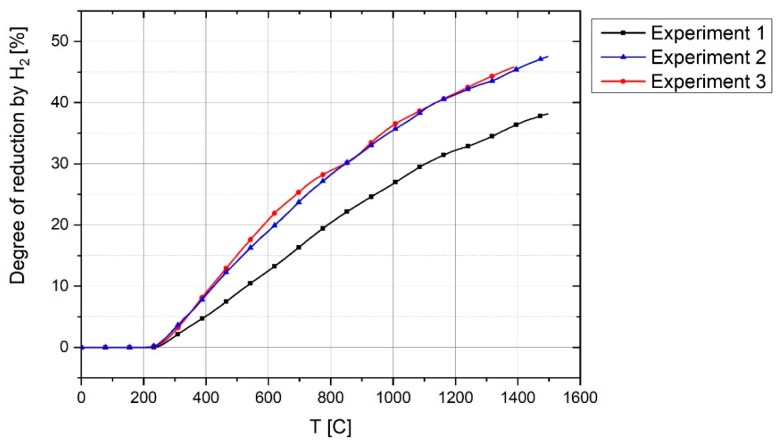
The degree of reduction of iron oxide in Experiments 1, 2 and 3.

The degrees of reduction of iron oxide considering only hydrogen are shown in Figure 16. At 1400 s, the degree of reduction by hydrogen for Experiments 1, 2 and 3 was 37%, 46% and 47% respectively. The main reason for the low degree of reduction is the use of the steel crucible, which not only caused the melt to freeze but also caused the arc to run in the previously reduced sections. Therefore, it is expected to find a higher degree of reduction if using refractory linings in the pilot plant. In the plasma reactor of the pilot plant or at industrial scale, all of the iron ore is in the liquid state, which has the benefit of achieving a reduction rate in the liquid state which is much higher than that of the solid state [17].

The chemical composition of the reduced iron was analysed to evaluate the reduction of other oxides and the behaviour of phosphorus. Table 8 shows the chemical composition of the iron produced using hydrogen.

From the comparison of the chemical composition of the iron ore (Table 3) and the reduced iron (Table 8), no reduction of silicon and alumina oxides could be observed. In terms of the basicity, the B_2_ of the slag was two, which caused a decrease of the phosphorus level from 0.06% to 0.01%.

**Figure 17:**
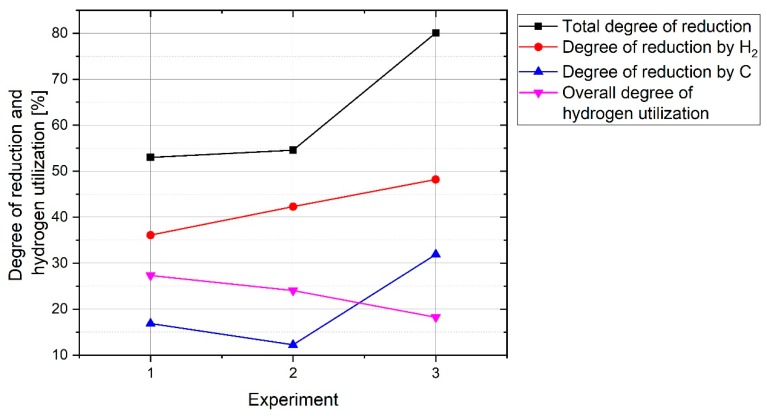
Degree of reduction and hydrogen utilization of Experiments 1, 2 and 3.

The total degree of reduction and degree of reduction by H_2_ and C, and the overall degree of hydrogen utilization were calculated, and the results are shown in Figure 17. To be comparable the results with each other, the calculations were done in a time of 1387 s operations which was the operation time of Experiment 3. It was stopped after this period own to the low amount of $\eta_{H_{2}}$.

The total degree of reduction and degree of reduction by H_2_ of Experiment 3 was higher than that of the two other experiments due to the lower weight of the sample. With a decrease in the sample weight, the degree of reduction by H_2_ increased. However, the contribution of carbon to the reduction reactions was different from experiment to experiment. The contribution of carbon to Experiment 2 was less than the others. The overall $\eta_{H_{2}}$ decreased by a decrease in sample weight consistently. The overall $\eta_{H_{2}}$ of Experiment 1 was approximately 27.3% which was less than the Gilmore MIDREX plant [33], because MIDREX is a continuous process with a constant flow rate of feed gas and feeding rate of iron pellets. However, the experiments were conducted in a batch reactor. In the beginning of the process, $\eta_{H_{2}}$ increased up to 60% and then decreased due to the reduction of iron oxides in the reduction zone. Therefore, if iron ore is continuously fed to the reactor, it is expected to have a $\eta_{H_{2}}$ more than 36.5% during the operation. For instance, for the operation between 300 and 800 s of Experiment 1, $\eta_{H_{2}}$ was greater than 36.5%.

Based on the degree of reductions, the amount of produced iron and slag were calculated. The results are shown in Table 9.

The reason for the production of a low amount of reduced iron by Experiment 2 was that the degree of reduction by carbon was less than the two other experiments. The composition of slags for the three experiments are shown in Table 10.

The FeO content in the slags was high and with an increase of the degree of reduction, the content decreased. The calculations show that phosphorous was captured in the slag because the amount of phosphorous in the produced iron was considerably less than that of in the iron ore.

## 5. Conclusions

This study has introduced the characteristics of the smelting reduction of haematite fine ore using hydrogen thermal plasma. The main parameters of the reduction process, namely the degree of hydrogen utilization, the reduction rate and the degree of reduction of haematite, have been assessed by conducting a series of experiments with different sample weights. Thermodynamic equilibrium calculations of the reduction of haematite in different aspects were carried out using FactSage^TM^ 7.2.

The degree of hydrogen utilization rose by 60% at the beginning of the experiments, and during the reduction process, owing to the diminishing of iron oxides, it then decreased. The running of the arc between the side of the crucible and the electrode caused the degree of hydrogen utilization to fluctuate. It is expected to reach a higher $\eta_{H_{2}}$ at pilot plant or industrial scale than was observed in these experiments at laboratory scale. This is because, at laboratory scale, owing to the use of the steel crucible and the low power of the electric supply, only the arc area was melted, and the remaining sections remained in solid state. 

The experimental setup and the use of a graphite electrode caused carbon to contribute to the reduction reactions. The reduction of haematite with the use of a graphite electrode meant that the contribution of carbon to the reduction process was 30% of the total degree of reduction.

From the assessment and comparison of the chemical composition of the produced iron and Carajas iron ore, the phosphorus content in the produced iron decreased by 0.01% due to the high basicity of the slag.

## Figures and Tables

**Table 1:** Inlet gas and off-gas composition of Gilmore plant.

Element	Unit	Inlet Gas	Off-Gas
H_2_	wt.%	52.58	37
CO	wt.%	29.97	18.9
H_2_O	wt.%	4.65	21.2
CO_2_	wt.%	4.8	14.3
CH_4_+N_2_	wt.%	8.1	8.6

**Table 2:** Experimental program.

Experiment	Sample Weight (g)	Total Gas Flow Rate (L/min)	H_2_/Ar Ratio (molar %)	B_2_ (CaO/SiO_2_)
1	100	5	50/50	2
2	75			
3	50			

**Table 3:** Chemical composition (wt.%) of Carajas iron ore.

No.	Element	(wt.%)
1	Fe(III) oxide	92.83
2	Fe(II) oxide	1.07
3	Total Fe	65.81
4	Silica	1.694
5	Aluminium oxide	1.01
6	Manganese (II) oxide	0.22
7	Manganese	0.17
8	Calcium oxide	0.01
9	Magnesium oxide	0.01
10	Phosphorus (V) oxide	0.131
11	Phosphorus	0.057
12	Sodium oxide	0.019
13	Carbon	0.098
14	Zinc	0.004
15	Sulphur trioxide	0.035
16	Total sulphur	0.014
17	Potassium oxide	0.017
18	LOI ^1^	2.79

^1^ Loss of ignition.

**Table 4:** Grain size distribution of Carajas iron ore and lime.

Mesh Size (mm)	Fraction (wt.%)	Cum (wt.%)
0.063–0.125	34	34
0.025–0.063	60	94
0–0.025	6	100

**Table 5:** Element content (wt.%) of ignition pin (IP) and steel crucible (SC).

Element	Unit	C	Si	Mn	P	S	Cr	Mo	Ni	Al	Cu
Composition of SC	(wt.%)	0.178	0.261	1.325	0.009	0.005	0.083	0.031	0.168	0.027	0.179
Composition of IP	(wt.%)	0.441	0.217	0.85	0.008	0.028	0.985	0.162	0.085	0.021	0.116

**Table 6:** Chemical composition (wt.%) of the calibration gas.

Component	H_2_	Ar	CO	CO_2_
Nominal concentration (wt.%)	29	63	6	2
Relative uncertainty	±1%	±1%	±1%	±2%
Actual concentration (wt.%)	29 ± 0.29	63 ± 0.63	6 ± 0.06	2 ± 0.04

**Table 7:** Deviation factor of each element.

Element	H_2_	Ar	CO	CO_2_
Unit	(wt.%)	(wt.%)	(wt.%)	(wt.%)
Deviation	−0.5	+0.35	+0.15	0

**Table 8:** Element content (wt.%) of reduced iron, Experiment 1.

Element	Fe	C	Mn	P	S	Cr	Mo	Ni	Cu
Concentration (wt.%)	99.7	0.004	0.05	0.01	0.03	0.03	0.04	0.05	0.05

**Table 9:** Produced iron and slag for operations in 1387 s.

Product Characteristics	Unit	Experiment 1	Experiment 2	Experiment 3
Produced iron	(g)	20.56	16.5	22.72
Metallization	(wt.%)	32.3	34.5	71.3
Slag weight	(g)	62.08	45.15	14.9

**Table 10:** Chemical composition of slags of Experiments 1, 2 and 3.

Element	Unit	Experiment 1	Experiment 2	Experiment 3
CaO	(wt.%)	5.17	5.32	10.7
MgO	(wt.%)	0.016	0.016	0.032
SiO_2_	(wt.%)	2.64	2.72	5.5
Al_2_O_3_	(wt.%)	1.56	1.62	3.28
FeO	(wt.%)	82.74	82.5	72.97
Fe_2_O_3_	(wt.%)	7.29	7.27	6.43
K_2_O	(wt.%)	0.026	0.027	0.055
MnO	(wt.%)	0.321	0.33	0.615
P	(wt.%)	0.2	0.207	0.41
S	(wt.%)	0.012	0.012	0
